# The Role of the Stromal Extracellular Matrix in the Development of Pterygium Pathology: An Update

**DOI:** 10.3390/jcm10245930

**Published:** 2021-12-17

**Authors:** Javier Martín-López, Consuelo Pérez-Rico, Selma Benito-Martínez, Bárbara Pérez-Köhler, Julia Buján, Gemma Pascual

**Affiliations:** 1 Departamento de Medicina y Especialidades Médicas, Facultad de Medicina y Ciencias de la Salud, Universidad de Alcalá, 28805 Alcalá de Henares, Spain; javier.martinlopez@gmail.com (J.M.-L.); selma.benito@uah.es (S.B.-M.); barbara.perez@uah.es (B.P.-K.); mjulia.bujan@uah.es (J.B.); 2 Departamento de Cirugía, Ciencias Médicas y Sociales, Facultad de Medicina y Ciencias de la Salud, Universidad de Alcalá, 28805 Alcalá de Henares, Spain; cinta.perezrico@gmail.com; 3 Biomedical Networking Research Centre on Bioengineering, Biomaterials and Nanomedicine (CIBER-BBN), 28029 Madrid, Spain; 4 Ramón y Cajal Health Research Institute (IRYCIS), 28034 Madrid, Spain

**Keywords:** ocular surface disease, pterygium pathology, extracellular matrix disorders, collagen, elastin

## Abstract

Pterygium is a benign fibrovascular lesion of the bulbar conjunctiva with frequent involvement of the corneal limbus. Its pathogenesis has been mainly attributed to sun exposure to ultraviolet-B radiation. Obtained evidence has shown that it is a complex and multifactorial process which involves multiple mechanisms such as oxidative stress, dysregulation of cell cycle checkpoints, induction of inflammatory mediators and growth factors, angiogenic stimulation, extracellular matrix (ECM) disorders, and, most likely, viruses and hereditary changes. In this review, we aim to collect all authors’ experiences and our own, with respect to the study of fibroelastic ECM of pterygium. Collagen and elastin are intrinsic indicators of physiological and pathological states. Here, we focus on an in-depth analysis of collagen (types I and III), as well as the main constituents of elastic fibers (tropoelastin (TE), fibrillins (FBNs), and fibulins (FBLNs)) and the enzymes (lysyl oxidases (LOXs)) that carry out their assembly or crosslinking. All the studies established that changes in the fibroelastic ECM occur in pterygium, based on the following facts: An increase in the synthesis and deposition of an immature form of collagen type III, which showed the process of tissue remodeling. An increase in protein levels in most of the constituents necessary for the development of elastic fibers, except FBLN4, whose biological roles are critical in the binding of the enzyme LOX, as well as FBN1 for the development of stable elastin. There was gene overexpression of TE, FBN1, FBLN5, and LOXL1, while the expression of LOX and FBLN2 and -4 remained stable. In conclusion, collagen and elastin, as well as several constituents involved in elastic fiber assembly are overexpressed in human pterygium, thus, supporting the hypothesis that there is dysregulation in the synthesis and crosslinking of the fibroelastic component, constituting an important pathogenetic mechanism for the development of the disease.

## 1. Introduction

**Figure 1:**
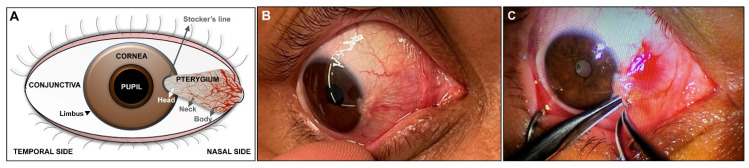
Surgical procedures. (**A**) Scheme of the pathology of a unipolar pterygium developing on the nasal side of the conjunctiva. Different areas in the anatomy of the eye and pterygium have been identified; (**B**) preoperative appearance in a grade II pterygium patient that exceeds the limbus and approaches the pupillary area; (**C**) beginning of the surgical process of pterygium excision in the cornea.

Pterygium is a benign fibrovascular lesion of the bulbar conjunctiva, which is related to chronic sun exposure, with frequent involvement of the corneal limbus that can invade the cornea. It usually shows a triangular wing shape, with the vertex opposite the base and directed toward the pupil and the base more frequently located in the nasal area toward the caruncle (Figure 1), although it can also arise from the temporal region. Pterygium can be unipolar if it affects only the nasal or temporal area of the conjunctiva or bipolar if it affects both. Similarly, it can develop in a single eye or appear bilaterally.

## 2. Clinical Diagnosis and Histopathological Characterization of Pterygium

Mild cases are usually asymptomatic; however, as the process progresses, it can cause symptoms in the form of redness, dry eyes, irritation, changes in ocular refraction, and vision problems. If left untreated, symptoms may increase in severity over time and may lead to significant vision loss due to infiltrative corneal growth.

Three parts of pterygium are defined: head, neck, and body (Figure 1). The head is a gray, flat, and avascular area at the apex. A pigmented line called the Stocker’s line is located at its anterior border, and it is associated with long-standing cases. The neck connects the head and the body, where finely branched neovessels are located. The body is located in the bulbar conjunctiva with vessels straight and radial to the apex. Generally, the head is firmly attached to the cornea, while the body can be separated from the anterior ocular surface. 

One of the clinical classifications of pterygium is based on the extent to which it covers the corneal surface. Thus, it is possible to distinguish between four grades: grade I, invades the corneal limbus; grade II, exceeds the limbus and approaches the pupillary area; grade III, reaches the pupil; and grade IV, exceeds the pupil.

Tan et al. [1] morphologically classified pterygium into three categories: atrophic, fleshy, and intermediate. In the atrophy category, episcleral vessels below the pterygium body are easily distinguished. In the fleshy category, pterygium shows a greater thickness so that the episcleral vessels below the body are not visualized. In the intermediate category, the vessels can be seen with difficulty.

**Figure 2:**
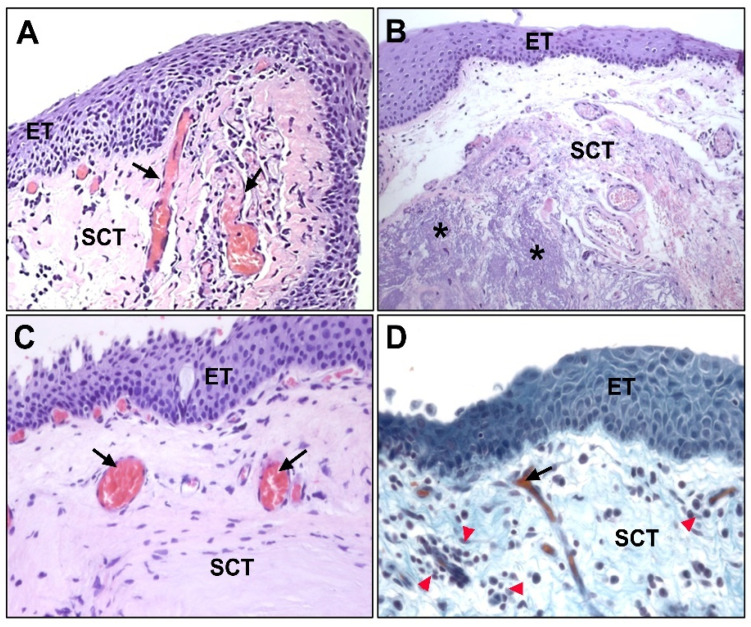
(**A**) Hematoxylin and eosin-stained image of healthy conjunctival tissue (×100); (**B**) amorphous and fibrillar material (*) can be observed in the subepithelial zones of pterygium (×100); (**C**) no amorphous or fibrillar material can be observed in normal conjunctival tissue (×200); (**D**) presence of lymphocytic infiltrate (►) near the vascular vessels in the subepithelial connective tissue of pterygium (×200). (ET, epithelial tissue; SCT, subepithelial connective tissue; →, blood vessels).

In the histopathological characterization of pterygium, the epithelial tissue does not present significant differences with respect to healthy conjunctiva. It usually shows varying degrees of acanthosis or alterations in keratinization in the form of parakeratosis or hyperkeratosis. On the contrary, the stroma is classically described as a thickening of the connective tissue, and it is characterized by elastotic changes in the thickness of the subepithelial stroma and associated lymphocyte-predominant inflammation (Figure 2) with respect to healthy conjunctiva. Thus, immature or fragmented elastic fibers are observed together with collagen fibers of variable thicknesses and mature-looking lymphocytes together with some scattered macrophages in the tissue.

In the subepithelial tissue of pterygium, large areas of extracellular matrix (ECM) with fibrillar and amorphous material can be observed, which are not observed in healthy conjunctiva. These areas do not have an affinity for eosin or for Masson’s trichrome light green dye, thus, discarding their collagenous nature. These areas show some basophilia or appear without evident staining, and they are identified based on elastotic alterations (Figure 2). In the subepithelial tissue, angiogenesis is very evident, and in the stromal tissue, a large number of blood vessels are observed. The lymphatic vessels are also very patent, dilated, and numerous.

## 3. Epidemiology and Pathogenesis of Pterygium

There is a higher prevalence of pterygium development in countries near the equator, i.e., up to 22% of the general population, whereas 2% prevalence has been estimated in populations from other latitudes [2]. A recent study with a total of 415,911 participants from 24 countries showed that the prevalence of pterygium in the total population was 12%. The lowest and highest prevalence rates were 3% in the 10- to 20-year-old group and 19.5% in those over 80 years old, respectively, and a similar prevalence was observed in men and women [3]. 

**Figure 3:**
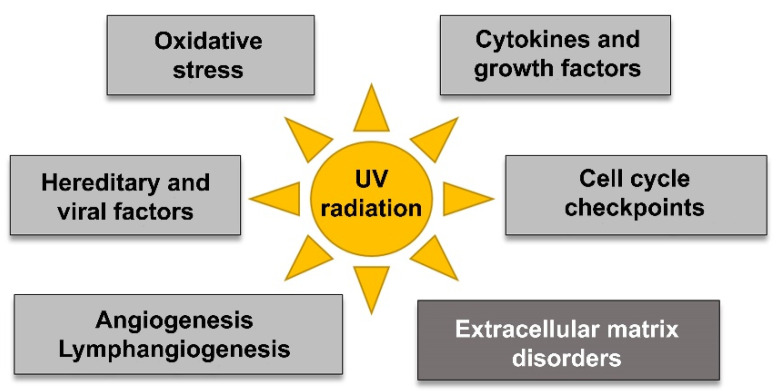
Summary of the multifactorial pathogenesis of pterygium.

This pathology is more frequent in outdoor workers, and its prevalence tends to increase with age [4]. The pathogenesis of pterygium has been fundamentally attributed to damage related to sun exposure to ultraviolet-B radiation (UV-B, wavelength, 280–320 nm), although evidence has been obtained that shows it is a complex process that includes multiple mechanisms (proinflammatory or immunological modifiers of ECM, cell proliferation and survival, or proangiogenic), the release of mediators (growth factors or cytokines), and probably viruses and genetic factors [5] (Figure 3).

### 3.1. Oxidative Stress 

Chronic solar exposure causes oxidative stress, which activates growth factors related to the development of pterygium. Oxidative stress is produced by an imbalance between reactive oxygen species (ROS), which include oxygen ions, peroxides, and free radicals, and a tissue’s capacity to reduce these species and repair the tissue damage that causes oxidative stress. The release of peroxides and free radicals is responsible for alterations of DNA, protein structure, and lipoperoxidation. The presence of 8-oxo-2′-deoxyguanosine, one of the classic markers of oxidative stress, has been described in pterygium samples by multiple authors [6,7].

### 3.2. Dysregulation of Cell Cycle Checkpoints 

In the pathogenesis of pterygium, a relationship with apoptotic regulatory mechanisms that condition its formation, growth, and persistence has been described. DNA fragmentation has been demonstrated by terminal deoxynucleotidyl transferase dUTP nick end labeling (TUNEL) marking, in addition to increases in antiapoptotic proteins Bcl-2 and BAX [8], as well as survival of apoptosis inhibitor [9]. Thus, chronic sun exposure has been correlated with oxidative stress and the expression of these antiapoptotic mediators.

However, most studies on the pathogenesis of pterygium have focused on describing alterations in cell cycle control points, such as p16, p53, p27, and cyclin D1, or on the state of loss of heterozygosity that has been described more frequently than microsatellite instability type [10]. In relation to cell cycle checkpoints, various authors have identified increases in p53 [11], p16 [12], as well as p27 and cyclinD1 [13], although they do not represent the mechanism underlying the presence of a somatic mutation in the TP53 gene [14,15], for which they associate an increase in its expression with the activation of these factors via intracellular signaling pathways.

### 3.3. Induction of Inflammatory Mediators and Growth Factors 

The vast majority of studies on the pathogenesis of pterygium have also described that the above alterations triggered a response that involved inflammatory mediators and growth factors that enhanced inflammatory and angiogenic responses. In this way, increases in the interleukins IL-1, IL-6, and IL-8 [16] and the tumor necrosis factor TNF-α [17] have been described as contributing to the recruitment of other inflammatory mediators and metalloproteases involved in pterygium pathogenesis.

However, the role of numerous growth factors in pterygium pathogenesis has also been described, such as heparin-binding epithelial growth factor (HB-EGF) [18], vascular endothelial growth factor (VEGF) [19], transforming growth factor β (TGF-β), platelet-derived growth factor (PDGF), and basic fibroblast growth factor (bFGF) [20].

### 3.4. Angiogenic Stimulation 

Angiogenesis research has been extensively analyzed in the pathology of pterygium. Inflammation promotes angiogenesis as an additional mechanism for the repair of tissue damage from inflammatory mediators and growth factors, especially VEGF, and the reduction of thrombospondin-1 [21]. VEGF promotes endothelial migration and is related to one of the classic mechanisms that promotes angiogenesis, such as in nitric oxide-rich cellular microenvironments, through the activity of endothelial nitric oxide synthase (eNOS) and inducible nitric oxide synthase (iNOS) [22]. Our research group has shown that the formation of new blood vessels was the most relevant event, and it was correlated with increased expression of vascular endothelial CD31 and an elevated blood/lymphatic vessel ratio. The presence of high levels of VEGF-A in both vessel networks and ECM in human pterygium tissue may have a major impact on angiogenesis in this pathological tissue [23].

### 3.5. Viruses and Hereditary Changes

Due to the influence of human papilloma virus (HPV) serotypes in various conjunctival pathologies (squamous papilloma and a subgroup of dysplasias and squamous carcinomas), its role in the proliferation of pterygium has been hypothesized, with discrepancies in the geographic distribution and serotypes described by different authors. However, a clear pathogenic association between pterygium and HPV- or herpes simplex (HSV)-type viral infections has not been established [24,25]. Viruses encode proteins that inactivate p53, which leads to chromosomal instability and increases the likelihood of cell progression to malignancy, although its implication remains controversial.

Moreover, specific hereditary traits involved in the pathogenesis of pterygium have not been described, and little evidence of family association has been observed. However, some authors have suggested that there could be an autosomal dominant inheritance pattern with incomplete penetrance [5]. Few studies have analyzed hereditary factors, and in most cases, the influence of an environmental or occupational factor is not ruled out before considering genetic alterations [2].

### 3.6. Extracellular Matrix Disorders

The ECM is a group of extracellular components secreted by stromal cells that provide structural and biochemical support to the cellular environment. Aberrant expression of ECM proteins may be directly associated with proliferative growth of pterygium. Tissue damage from chronic sun exposure and the activation of inflammatory mediators increase the expression of matrix metalloproteases (MMP-1, MMP-2, MMP-3, MMP-7, MMP-8, MMP-9, MMP-14, and MMP-15), which leads to modification/remodeling of the ECM [26,27]. These alterations may be an initial change in the development of pterygium at the level of the limbus in which the components of the stromal connective tissue, elastin, and tropoelastin (TE) are altered [28]. The fibrovascular tissue that makes up pterygium is characterized by an increase in elastin and myofibroblasts, which plays a critical role in the migration and growth of pterygium [29].

Due to the scarcity of studies related to the latter mechanism implicated in the development of pterygium pathology, in this study, we focus on ECM disorders and review the most studied ECM constituents, with a special emphasis on updating and summarizing the main findings obtained by our research group, whose members have many years of experience in the study of the collagen and elastic components of different soft tissues, including pterygium. 

## 4. Role of ECM in Tissue Repair and Pathological Processes

ECM is a coordinated network composed of multiple molecules that make up a three-dimensional structure with physical properties that play a fundamental role in cell adhesion, structure, and tissue and organ support. However, the interconnection of these molecules and their functional interactions represent microenvironmental signals that influence cell differentiation, proliferation, survival and migration.

ECM also has a dynamic role in physiological mechanisms, such as tissue repair or healing, or in pathological contexts, such as cancer, in which ECM changes are induced by multiple mediators and growth factors, which condition various effects, such as stimulation of angiogenesis and inflammatory responses and promotion of stromal invasion that can lead to an excessive accumulation of proteins or differentiation of cellular components. 

There are multiple cells that collaborate in the promotion of an unstructured matrix, such as endothelial cells, pericytes, cancer-associated fibroblasts (CAFs), and immune cells. One of the mechanisms identified is an increase in the activity of the lysyl oxidase (LOX) enzymes, which promotes crosslinking of collagen and its interaction with ECM components and increases rigidity [30].

The enzymes responsible for the degradation of ECM are MMPs, hyaluronidases, disintegrins, ADAMs, ADAMTS, as well as plasminogen activators and proteases such as granzymes and intracellular cathepsins. The degradation of the ECM coexists with the production of new elements and their accumulation. Fibroblasts are the main source of matrix components, although remodeling is a process involving multiple cells. The alteration of normal remodeling is an initiating factor in pathological processes and their progression.

Fibroblasts are involved in the synthesis of ECM components, and they can acquire contractile capacity and can participate in the secretion of cytokines and matrix mediators. They play a fundamental role in tissue repair and healing processes, in which activated fibroblasts produce myofibroblasts through the expression of α-smooth muscle actin (α-SMA) filaments mediated by the activation of the SMAD2 protein.

Fibroblasts participate in the pathogenesis of pterygium via their activation to myofibroblasts, their secretion of mediators and their interactions with other ECM elements. The magnitude of tissue damage and aberrations in the activation and functionality of fibroblasts, either in their proliferation, production of collagen or elastic fibers, and migration or differentiation to myofibroblasts, are among the mechanisms involved in the alteration of tissue repair and the pathological processes of ocular fibrosis.

## 5. ECM and Its Pathogenic Mechanisms in the Development of Pterygium

In the pathogenesis of pterygium, epithelial cells are proposed to be responsible for an alteration in the balance between proliferation and apoptosis, which conditions a stromal overgrowth of activated fibroblasts, thereby, promoting angiogenesis, inflammation, and aberrant elastin and collagen accumulation in ECM. Furthermore, pterygium epithelial cells show characteristics involved in the epithelium-mesenchymal transition, such as the loss of E-cadherin and the nuclear accumulation of β-catenin [31]. Other models of epithelial-mesenchymal transition from epithelial cells have shown how the expression of epithelial markers is reduced and the expression of mesenchymal markers increases [32]. Phenotypic changes induce morphological changes in cell interactions and functions. Among the mechanisms described are the change from E-cadherin to *N*-cadherin and the expression of α-SMA or other mesenchymal markers or transcription factors, such as vimentin, FSP-1 (fibroblast specific protein 1), Snail, Slug, TWIST, and ZEB1 [33]. Thus, it has been postulated that myofibroblasts are derived from keratinocytes [34], progenitor cells of the limbus [35], orbital fibroadipose tissue [36], or cells from bone marrow [37].

Elevated levels of TGF-β expression have been reported in pterygium samples [20] and in cultures of isolated pterygium fibroblasts [38]. Antifibrotic treatments in other organs have led to studies that evaluated the efficacy of such treatments, for example, the expression of TGF-β in cultured pterygium fibroblasts has been inhibited, and a decrease in cell proliferation, migration, and collagen synthesis has been observed [39]. Treatment with human amniotic membrane grafts suppresses the expression of TGF-β2, TGF-β3, and TGFBR receptors in cultured pterygium fibroblasts, with the consequent inhibition of contractility [40]. Moreover, a reduction in α-SMA expression in cultured pterygium fibroblasts [41] has led to improved healing.

A number of studies have relatively frequently reported the role of other ECM components in pterygium not related to fibroblasts or TGF-β, such as MMPs [29], different growth factors (PDGF, bFGF, HB-EGFM, and VEGF) [18,38], or inflammatory mediators, such as IL-6 and IL-8 [42].

The activities of various enzymes, such as cyclooxygenases (COX), lipoxygenases, or cytochrome P450, have also been described in relation to increases in proinflammatory mediators [43], although the expression of LOX has not been characterized in relation to processes such as elastogenesis.

In the field of ophthalmological research, alterations in elastogenesis have been evaluated mainly in corneal diseases, such as macular degeneration with respect to fibulins (FBLNs) or fibrillins (FBNs) [44,45], in the dysfunction of LOX-like 1 (LOXL1) action in glaucoma models related to exfoliation syndrome [46,47], or in keratoconus [48].

Experimental studies of pterygium in which alterations in essential components for elastogenesis have been characterized are scarce [49] and have not described alterations in the expression and functionality of TE, LOXs, or proteins of the family of FBLNs or FBNs.

As our research group is a pioneer in the analysis of the elastic component in the pathogenesis of pterygium, all the results obtained by our group about alterations found exclusively at the level of the fibroelastic component of pterygium are shared below, with special emphasis on the constituents and the assembly and reticulation process of the elastic fiber.

## 6. Fibroelastic Alterations in Pterygium ECM

The ECM of pterygium includes fibrillar elements, such as collagens and elastic fibers and an amorphous component (proteoglycans, multi-adhesive glycoproteins, and glycosaminoglycans) that constitutes the ground substance.

These components interact in a complex way with each other as well as with other elements of the matrix and various cell types (such as endothelial, immune, or epithelial cells). Interactions occur through surface receptors, such as integrins, discoidin domain receptors (DDRs), cell surface proteoglycans (such as syndecans), and hyaluronan receptors (such as CD44). In addition, they interact with different growth factors and with MMP enzymes that maintain the integrity and remodel the composition of the ECM.

In this case, we focus on the in-depth analysis of the two main fibrillar elements of the ECM, collagen fibers (types I and III), as well as the main constituents of elastic fibers (TE, FBNs, and FBLNs), and the enzymes (LOXs) that carry out their assembly or crosslinking.

### 6.1. Collagen

Collagen is the most abundant component of the ECM, and it is also present in pericellular regions. It is synthesized from fibroblasts, which also have a role in its spatial arrangement and organization. Collagen is formed from three polypeptide chains called alpha chains, which can be organized to create homodimeric or heterodimeric triple helices. The α chains are formed from triplets of Gly-X-Y, with X and Y representing the amino acids proline and hydroxyproline, respectively. The triple helices crosslink to form crosslinked collagen fibrils in the ECM.

Fibrillar collagens are found in multiple tissues that confer tensile strength and are involved in cellular functions, such as cell migration and adhesion, angiogenesis, and tissue development and repair.

In the eye, the cornea is the anatomical structure with the greatest presence of collagen [50]. The corneal stroma accounts for 90% of the stroma and is composed of an abundant amount of collagen, especially type I, although the presence of multiple types of collagens has been identified, most at the stromal level (types II, III, V, XIII, etc.). Regarding the conjunctiva, the predominant collagen is type VII collagen at the level of the basement membrane, where it forms anchor fibrils, which have also been identified in the basement or Bowman membrane of the cornea or at the level of the limbus [51,52], and the predominant types in subepithelial connective tissue are I and III.

Our research group has carried out different studies to evaluate the expression of different types of collagens in pterygium tissue [53]. Through observations with polarized light, Sirius red staining has made it possible to jointly assess type I and III collagens and to identify the location and balance of both types in healthy conjunctiva and pterygium. This technique is based on the orientation and interaction between the sulfone groups of the dye and the amine groups of lysine and hydroxylysine and guanidine groups of arginine in the collagen fibers, and the colors differ depending on the degree of collagen maturity. Collagen type I (mature collagen) stains reddish orange whereas collagen type III (immature collagen) stains yellow–green.

**Figure 4:**
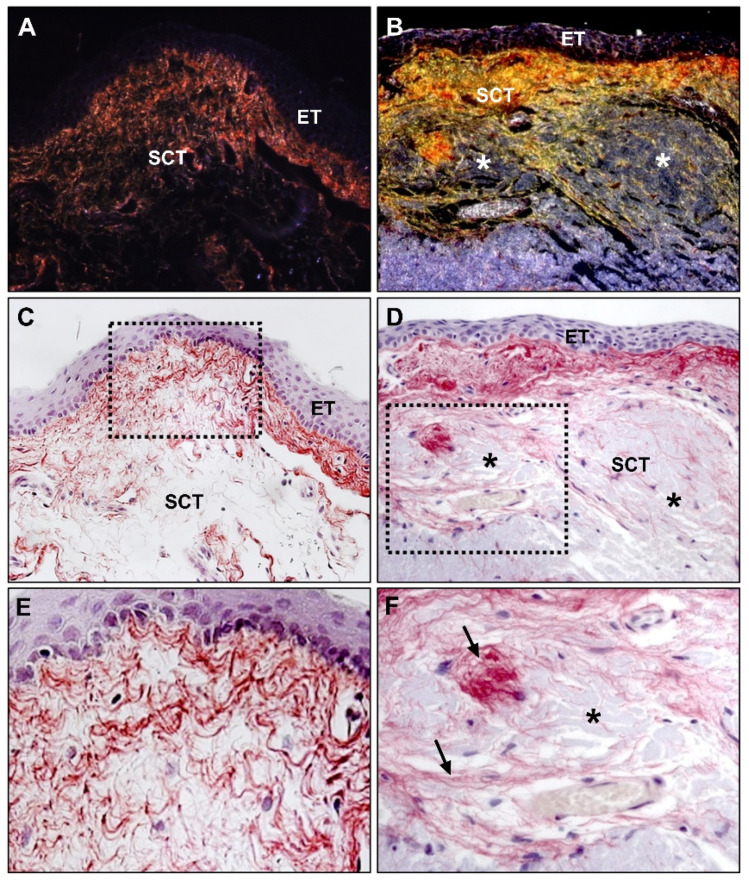
Photomicrographs of Sirius red staining observed under polarized light of (**A**) conjunctival and (**B**) pterygium tissue, showing expression of collagen I (mature) in red and collagen III (immature) in yellow, in the subepithelial connective tissue of both specimens (×200); (**C**) conjunctival and (**D**) pterygium tissue images of the same samples stained with Sirius red observed under normal light, where collagen expression appears in red (×200); (**E**,**F**) magnification of the squared area from the (**C**,**D**) image showing collagen fibers (→) (×400). (ET, epithelial tissue; SCT, subepithelial connective tissue; *, areas of amorphous and fibrillar material accumulation; →, collagen fibers).

The two types of collagens are located in the ECM of the subepithelial stromal tissue of both types of tissue samples. In healthy conjunctiva samples, collagens type I and III are present in similar proportions, while in pterygium samples, the most immature form of collagen (type III) is increased, thus, indicating a new process of synthesis and deposition of collagen and suggesting a process of tissue formation and remodeling (Figure 4). In deep areas, the collagen fibers infiltrate and distribute as a reticulum between the amorphous fibrillar areas of the pterygium samples. These areas with a fibrillar or amorphous component are not stained by Sirius red; thus, they appear without staining under the light microscope and with a translucent appearance under polarized light, which indicates that these structures do not have a collagenous nature, and therefore correspond to immature or fragmented elastic fibers (Figure 4).

### 6.2. Elastin and Elastogenesis

**Figure 5:**
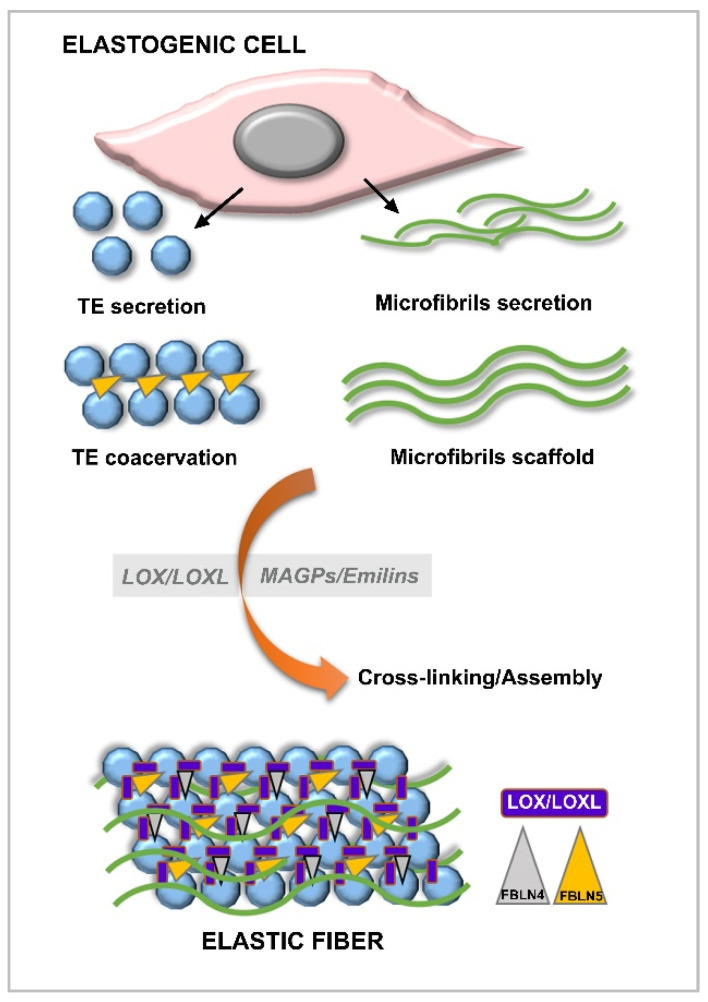
Process of elastogenesis and the molecular interactions among the different components of the elastic fiber. Functions of LOX/LOXL, fibulin-4 (FBLN4), and fibulin-5 (FBLN5) during coacervation, crosslinking, and assembly have been shown.

The elastic fibers of the ECM are formed by a compact network with two main components, with the majority represented by elastin together with a network of microfibrils of fibrillins [54]. Elastin is another structural protein closely related to collagen that provides elasticity to tissues and stability to ECM components. In its development and operation, TE, FBNs, FBLNs, LOXs, and other associated proteins are necessary (Figure 5).

Elastic fibers are assembled in developmental stages and represent stable structures [55]; however, tissue damage and pathological processes can cause their degradation by MMPs, which releases elastin fragments that promote monocyte chemotaxis and fibroblasts that will activate changes in the ECM together with incorrect repair and abnormal functioning of the fibers [56].

#### 6.2.1. Tropoelastin

Elastin is an insoluble polymer composed of monomeric subunits of tropoelastin TE that are crosslinked with a framework of fibrillin microfibrils that form elastic fibers. TE contains hydrophobic residues rich in valine and glycine that are responsible for the elastic properties of fibers, and it also contains other smaller lysine domains, the latter of which are modified by LOX or LOXL. Essential for the correct formation of elastin is its extensive crosslinking by LOX enzymes that oxidize selective lysine residues to align to form desmosine and isodesmosine crosslinks that stabilize the elastin polymer and render it insoluble.

However, in addition to elastin, elastic fibers are made up of various microfibrils whose main function is to form a necessary framework for the configuration of elastic fibers, including MAGP (microfibril-associated glycoproteins), LTBP (latent TGFβ binding protein), interface molecules, and especially, FBN1 (predominantly) and FBN2 glycoproteins [57,58] (Figure 5).

**Figure 6:**
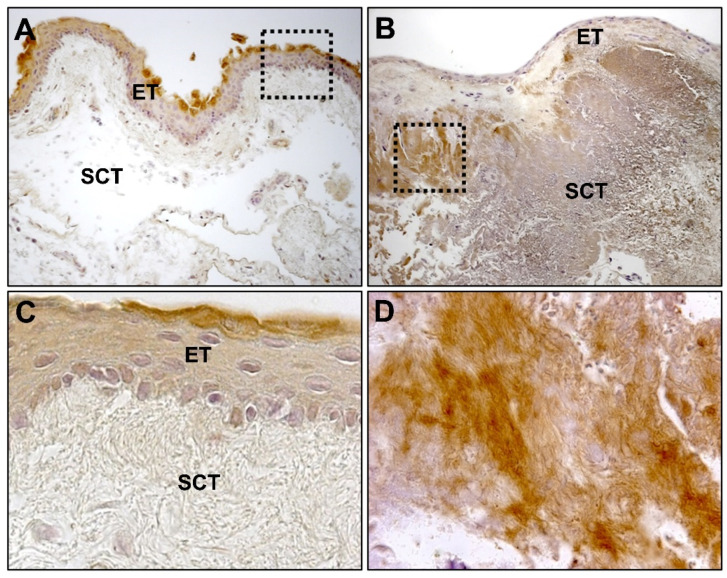
Images of immunohistochemical tropoelastin staining show an increased expression in pathologic tissue: (**A**) Conjunctival tissue (×100); (**B**) pterygium (×100); (**C**,**D**) detailed view of the squared section in (**A**,**B**), respectively (×630). (ET, epithelial tissue; SCT, subepithelial connective tissue).

An immunohistochemical analysis has been performed and revealed the presence of TE in the stroma of healthy conjunctiva, and showed how the expression of this elastic component was reduced. Large areas of low density were observed with minimal expression of TE and slight marking in some thin fibrillar elements of the ECM. Therefore, these results showed the predominance of the collagen component and nonfibrillar matrix over the elastic component in healthy conjunctiva. In contrast, the expression of TE was significantly increased in pterygium, where it was observed in the subepithelial tissue as large areas with degenerative changes or immature formations of elastic fibers. The labeling was located in the amorphous material and thickened and tortuous fibers of the subepithelial connective tissue (Figure 6).

**Figure 7:**
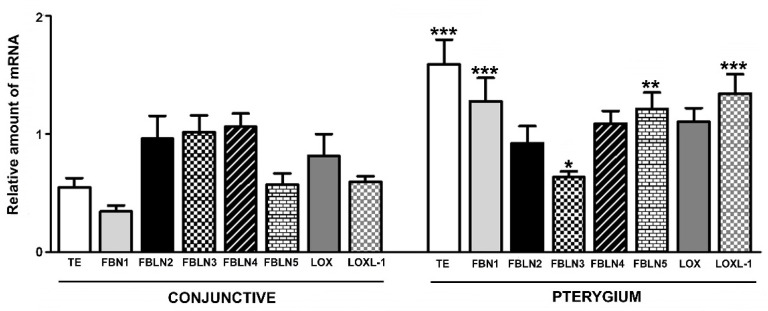
Relative quantification of tropoelastin (TE), fibrillin-1 (FBN1), fibulin-2 (FBLN2), fibulin-3 (FBLN3), fibulin-4 (FBLN4), fibulin-5 (FBLN5), LOX and LOXL1 messenger ribonucleic acid (mRNA) in conjunctival and pterygium tissue. Gene expression was normalized with glyceraldehyde 3-phosphate dehydrogenase (GAPDH). (* *p* < 0.05, ** *p* < 0.01, and *** *p* < 0.001).

The mRNA analysis results for TE correlated with the immunohistochemical findings and showed a significant increase (*p* < 0.001) in the pterygium group as compared with healthy conjunctiva, with gene expression increasing approximately 2.8 times in the active pterygium group (Figure 7). 

#### 6.2.2. Fibrillins

FBNs are extracellular glycoproteins that compose the microfibrils on which elastin is deposited, and they are located within and on the periphery of the elastic fiber. In addition to being the predominant component of the fibril framework of elastic fibers, they interact closely with TE and integrins. Three isoforms, FBN1, -2, and -3, have been described and are characterized by an amino acid region together with cysteine domains that bind TGF-β and calcium domains that bind EGF. While FBN2 and FBN3 are mainly expressed in the embryonic period, FBN1 appears in both embryonic and adult tissues. Mutations in the fibrillin genes cause alterations in elastogenesis and connective tissue disorder conditions, such as Marfan syndrome or Weill-Marchesani syndrome if mutations occur in the FBN1 gene, or congenital contractural arachnodactyly (Beals syndrome) if the FBN2 gene is altered.

**Figure 8:**
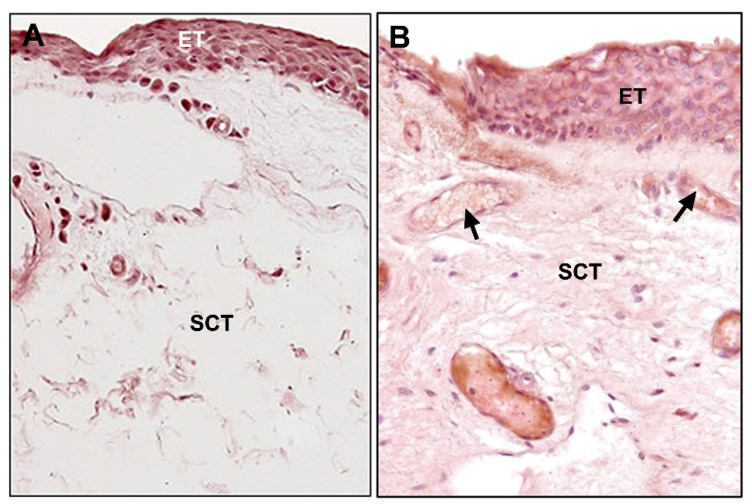
Photomicrographs show immunohistochemical staining for fibrillin-1: (**A**) Conjunctival tissue (×400); (**B**) pterygium (×400). Fibrillin-1 expression was increased in pathologic tissue. (ET, epithelial tissue; SCT, subepithelial connective tissue; →, blood vessels).

Very low levels of FBN1 have been observed in the stroma of healthy conjunctiva. However, the pathological population showed a significant increase in FBN1 immunostaining in the ECM (Figure 8). Gene expression for FBN1 has been revealed by quantitative PCR techniques and was also increased in this pathological population, where it was four times higher than that found in conjunctiva samples (*p* < 0.001) (Figure 7).

#### 6.2.3. Fibulins

Since the discovery of fibulin-1 [59], seven members of the FBLNs family have been described in the last 30 years [60,61], and they have been functionally characterized both in vitro and in physiological and pathogenic states. They are divided into class I and class II based on their length and the structure of their domains. Specifically, class II FBLNs (FBLN3, FBLN4, and FBLN5) behave as short FBLNs of the elastogenic type (due to the presence of a calcium domain that binds to EGF similar to that of FBN1), thus, exerting a fundamental role in the development of elastic fibers [62]. The most important biological role in elastogenesis corresponds to FBLN4 and -5. FBLN5 has a greater capacity to bind TE than FBLN4, and it also has a greater capacity to enhance the formation of elastic fibers. However, the biological role of FBLN4 in elastin development appears to be critical, because FBLN4 knockout animal models are lethal during gestation and the neonatal period [63,64,65], while FBLN5 knockouts are capable of living with progressively accumulating defects of the elastic fibers [66,67].

FBLNs are necessary for the assembly and function of elastin, and they are also capable of binding integrins and establishing cell and ECM interactions. For example, FBLN1 interacts with cytoskeletal proteins and has been identified around fibroblasts in in vitro and embryonic models [68]. FBLN2 is able to bind elastin to FBN1 and to participate in its anchoring to the fibrillin microfibril network, while FBLN3 interacts by binding elastic fibers to basement membranes.

In elastogenesis, the interactions of TE with FBLN4 and FBLN5 are critical for binding LOX enzymes and FBN1 and for forming stable elastin.

**Figure 9:**
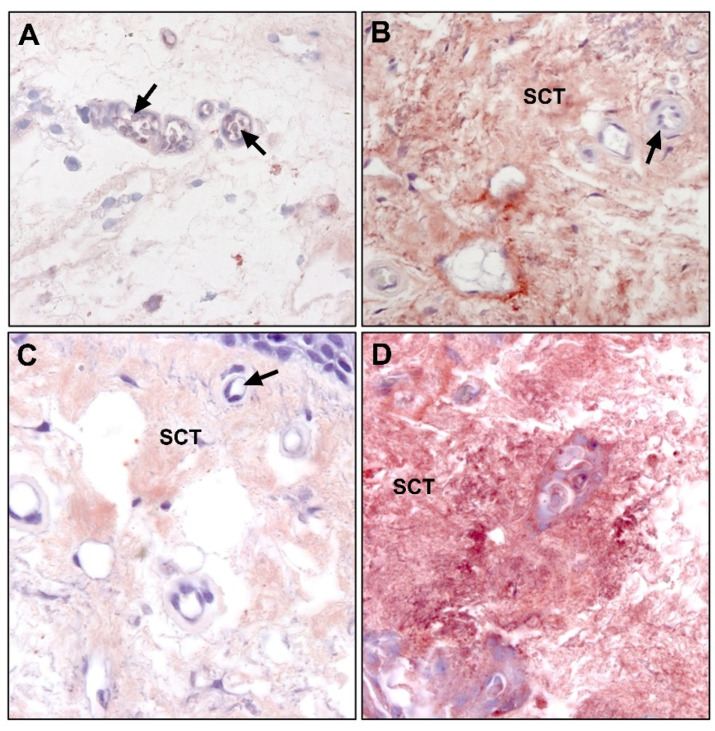
Expression of fibulin-2 localized in the subepithelial connective tissue in both (**A**) conjunctival and (**B**) pterygium tissue (×630); (**C**) fibulin-3 expression in conjunctival sample (×630); (**D**) positive labeling for fibulin-3 in pterygium tissue (×630). Higher expression levels of fibulin-2 and fibulin-3 were observed in pterygium with respect to the conjunctiva. (SCT, subepithelial connective tissue; →, blood vessels).

We have been pioneers in the analysis of the most important FBLNs in the development of elastic fibers (FBLN2, -3, -4, and -5). Our studies have shown that a significant increase in FBLN2 expression generally occurred in the subepithelial tissue of pterygium. Immunostaining in the stromal area occurred in the ECM, and it was relatively more intense around the blood and lymphatic vessels and in the areas of fibrillar or amorphous material accumulation (Figure 9A,B). As compared with TE, FBLN2 gene expression did not increase in the pathological samples as compared with healthy conjunctiva, with both groups presenting very similar values (*p* > 0.05) (Figure 7).

Our studies have also shown that healthy conjunctiva presented similar expression patterns for FBLN3 and FBLN2, with FBLN3 colocalizing with FBLN2, although a difference was observed in the more intense labeling in areas of the subepithelial connective tissue in contact with the basal epithelium. However, we found that the expression of FBLN3 in pterygium increased significantly and spread homogeneously throughout the subepithelial connective tissue; moreover, a significant increase in FBLN3 expression was observed in areas closer to the blood and lymphatic vessels (Figure 9C,D). The expression of mRNA in healthy patients was very similar to that of FBLN2; however, in pterygium, the expression was decreased approximately 1.5 times as compared with that of healthy samples (*p* < 0.05) (Figure 7).

**Figure 10:**
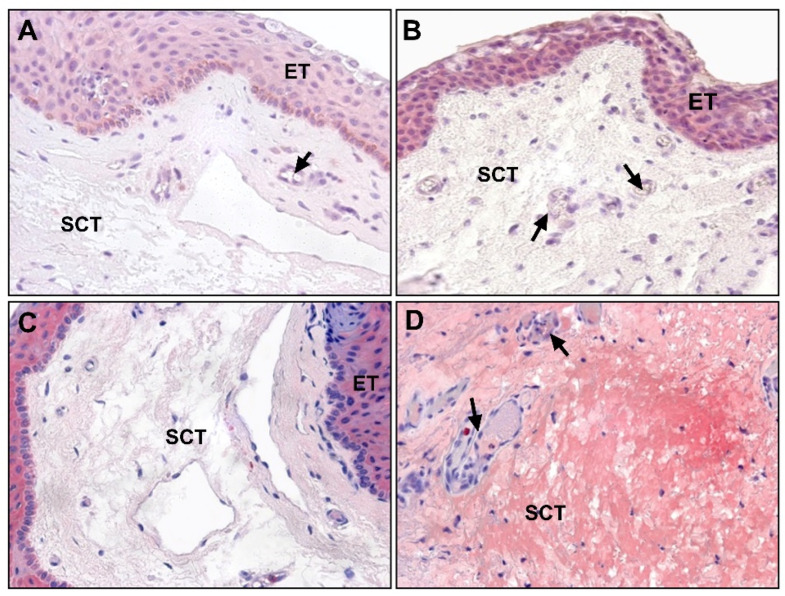
Fibulin-4 expression in (**A**) conjunctival and (**B**) pterygium samples (×400). Immunohistochemical staining of fibulin-5 expression in (**C**) conjunctival and (**D**) pterygium tissue (×400). Fibulin-4 expression was similar between the healthy and pathological groups. In contrast, increased expression of fibulin-5 was localized in the pterygium subepithelial connective tissue. (ET, epithelial tissue; SCT, subepithelial connective tissue; →, blood vessels).

The results of our immunohistochemical studies have shown that, contrary to FBLN2 and FBLN3, no differences were observed in FBLN4 protein expression between the healthy and pathological groups; both groups showed similar labeling in the subepithelial connective tissue, and the expression was very low (Figure 10A,B). Similar to the immunohistochemical study, no differences were found in the expression of the gene for FBLN4 and both study groups showed similar values for the relative amount of the messenger (Figure 7).

The subepithelial connective tissue shows weak immunolabeling for FBLN5 in healthy conjunctiva, while the levels are significantly increased in pterygium, which show very marked areas of degenerative elastogenic changes or immature fiber formation (Figure 10C,D).

In general, the mRNA levels of FBLN5 coincide with significantly higher protein expression, by approximately 2.5 times in a pathological population vs. healthy conjunctiva (*p* < 0.01) (Figure 7).

#### 6.2.4. Lysyl Oxydases

Lysyl oxidases (LOXs) are the enzymes responsible for the assembly of collagen and elastin, which form the desmosine bonds. They belong to a heterogeneous family of amino oxidases that oxidize the amino substrate to aldehyde. LOX and four isoforms of LOXL, (namely, LOXL1, LOXL2, LOXL3, and LOXL4) have been described as performing such oxidization, and they are synthesized in their inactive proenzyme form. They all share the C-terminal catalytic region in common and are differentiated by the *N*-terminal region. Its main substrates are collagen fibers and TE, oxidizing lysine or hydroxylysine residues into lysine or hydroxylysine for TE and collagen fibers, respectively. These aldehydes can react spontaneously to form the covalent bonds that confer resistance to collagen fibers and elasticity to elastic fibers. However, other more specific functions have been described for these enzymes, such as the possible roles they play in the control of cell adhesion and growth determined by domains such as the “cytokine-like” receptor domain [69].

Elastin crosslinking is another critical point for the synthesis of polymerized insoluble elastin. This process is mediated by the LOX family of enzymes, and in vitro models have shown that interactions occurred with proteins of the FBLN family [70]. In this way, FBLN4 mediated the binding of TE to LOX [71], while FBLN5 did so by interacting with LOXL1 [72,73]. Its role was critical in identifying lethality in LOX knockout models [74] due to rupture of the aorta and diaphragm due to incomplete elastin crosslinking.

**Figure 11:**
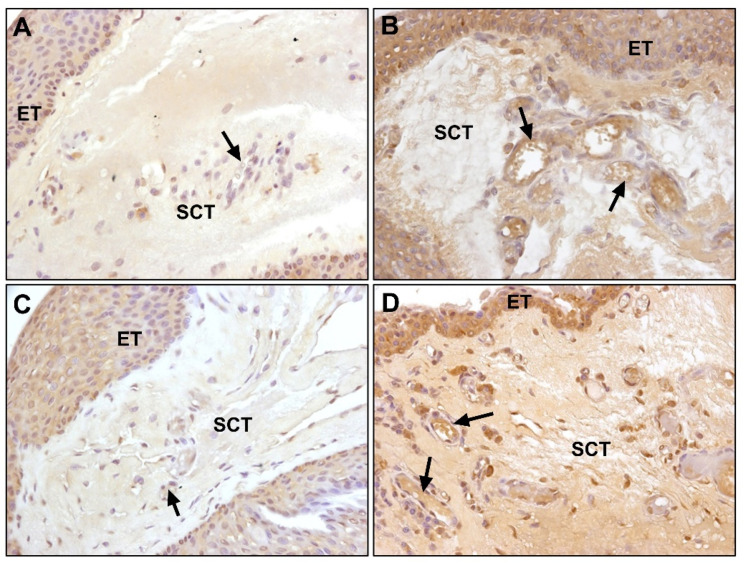
Immunohistochemical labeling for LOX in (**A**) conjunctival and (**B**) pterygium tissue (×400). LOXL1 expression in (**C**) conjunctival and (**D**) pterygium tissue (×400). LOX and LOXL1 can be observed in the subepithelial matrix in both samples, with a higher expression in pterygium. (ET, epithelial tissue; SCT, subepithelial connective tissue; →, blood vessels).

The protein expression of LOX showed immunostaining that appeared mainly in the ECM and was significantly higher in pterygium (Figure 11A,B). This result is perfectly justifiable given the participation of both enzymes LOX and LOXL in the crosslinking of collagen and elastin, forming complex crosslinks essential for the stabilization of collagen fibrils and for the integrity and functionality of mature elastin.

In contrast, the mRNA expression did not correlate with the protein expression and was not increased in pterygium (Figure 7).

Taking into consideration that FBLN4 is capable of binding to TE and FBN1, thereby, mediating the maturation and crosslinking of elastic fibers through strong LOX binding and directing the deposition of elastin in microfibrils, in pathological samples, the difference in protein expression between FBLN4 and LOX (which is increased) is not argued. This reduced expression of FBLN4 could be one of the factors associated with the development of elastotic alterations and the immature and fragmented elastic fibers observed in pterygium pathology.

Our experience shows that immunohistochemical labeling for LOXL1 presents an expression pattern very similar to that of LOX. It appears mainly in the subepithelial matrix in both samples, although higher expression is observed in the pathological samples. Given that LOXL-1 seems to be necessary more specifically in the crosslinking of TE that participates in the formation, maintenance, and remodeling of elastic fibers, particularly during dynamic processes, the increase of this protein in the ECM of pterygium would be totally justified (Figure 11C,D).

As compared with LOX, the relative amount of LOXL1 mRNA was correlated with protein expression and showed a significant increase of approximately two-fold in pterygium as compared with healthy conjunctiva (*p* < 0.001) (Figure 7).

This same pattern of expression and the increase in protein expression observed in the pathological samples of FBLN5 corresponded to the analysis of LOXL1. Considering that FBLN5 was capable of binding TE and FBN1 and mediated their assembly through its interaction with LOXL1 as well as promoted the aggregation of TE molecules by coacervation, LOXL1/FBLN5 colocalization has been fully proven. 

## 7. Discussion

Collagen and elastin, the main components of the ECM, are intrinsic indicators of physiological and pathological states. To understand healthy and diseased tissues in pterygium pathogenesis, an investigation of the modification of these main structural proteins is necessary, which is what we have tried to summarize in this review article. 

Due to the pathogenic relationship between chronic exposure to solar radiation and the development of pterygium, studies have focused on how this chronic exposure to solar radiaiton activates the expression of inflammatory mediators and cytokines [75] in addition to matrix metalloproteases [76] that produce conformational changes in the matrix components identified in pterygium samples. However, in addition to inflammatory mediators and metalloproteases, the expression of growth factors, such as HB-EGF [77], which is involved in tissue or bFGF healing processes, has been described, as reported in recurrent pterygium [38]. This inflammatory microenvironment leads to the cooperative activation of other growth factors and other pathogenic mechanisms, such as angiogenesis, as well as the activation and functionality of stromal fibroblasts [78], which acquire a myofibroblast phenotype involved in the activation of various signaling pathways, such as mTOR [79], which modifies the composition of the ECM. Previous studies have indicated that the basal cells of the limbus and stromal fibroblasts secrete TGF-β, and thereby synthesize elastic material [80], and produce several types of MMPs similar to those reported in tumor models [81].

Gene and protein expression studies are also optimal tools to elucidate the pathogenic mechanisms involved in the development of pterygium. The data reported based on microarray analyses have included the relationship of miR-125 with fibroblast proliferation and the production of ECM components [82], the influence of miR-218-5p on EGFR expression and its activity on the PI3K/AKT/mTOR signaling pathway involved in cell proliferation and migration [83], the influence of miR-21 on the PTEN/AKT pathway [84], and the influence of miR-143-3p, miR-181-2-3p, miR-377-5p, and miR-411a-5p on pterygium fibroblasts [85].

In addition to modifications at the promoter level, studies have shown that the pathogenesis of pterygium may be related to the DNA methylation state, which would imply alterations in the genes involved in the expression of proteins, such as CD24, MMP-2, or TGM-2, which play essential roles in wound healing and development [86]. Therefore, these epigenetic changes could determine the recurrence of lesions after surgery.

Characteristically, the histopathological description of pterygium has indicated a highly vascularized subepithelial stromal tissue with the presence of morphological changes in collagen and elastic fibers consisting of hypertrophic and elastotic fibers, respectively. Fibroblastic activation induced by sun exposure was initially postulated to first affect the correct configuration of the elastic fibers and to cause abnormal maturation, which is called elastodysplasia, and then lead to secondary degenerative changes, such as elastodystrophy [49].

These fibroelastic changes are not exclusive to the pathology of the ocular surface and are frequently identified at the level of the superficial dermis in the histopathology of actinic keratosis-type skin lesions or in skin carcinomas related to sun exposure [87,88]. 

In the eye, elastin, together with collagen, is one of the main stromal components of the cornea, the cribriform plate, and the peripapillary sclera, and both the epithelium and the corneal endothelium have been reported to synthesize fibrillar components involved in the synthesis of elastin. A decrease in precursor components in the final stages of life is characteristic [89] and has been related to the development of glaucoma [90,91].

Few models of ophthalmological diseases have been developed in which the influence of the degradation and configuration of elastic fibers on their development has been studied. One model is the pathogenesis of involutional ectropion and entropion, in which a significant loss of elastic fibers and overexpression of MMP-2, MMP-7, and MMP-9 has been identified [92].

In addition to vascular proliferation, one of the most obvious morphological characteristics of pterygium is the presence of these changes at the level of the elastic fibers; however, few studies have directly focused on the elastic component and changes that occurred around its configuration or degradation in the primary disease, such as in recurrences. 

For the proper configuration of the elastic matrix to occur, the monomeric form of elastin, TE, will develop multiple complex interactions with the entire series of associated proteins, which include FBNs, FBLNs, and matrix-associated glycoproteins (MAGPs) [93]. Therefore, TE is a common ligand for these proteins, and as observed in our studies, colocalization in their expression pattern occasionally occurs.

Our studies showed general increases in the TE, FBN1, FBLN5, and LOXL1 mRNA levels in pterygium as compared with in control conjunctiva, although this was not observed in the FBLN2, -3, -4, and LOX analyses. However, at the protein level, we identified an increase in all of their levels except for FBLN4 and the immature form of collagen.

Regarding the expression of TE, our results agree with those described by other groups [28] that have also found high levels of expression of this protein in pterygium. This may be the result of mutations in the untranslated region but not in the coding sequence of TE mRNA, which would lead to errors in DNA polymerase activity and a massive accumulation of abnormal elastic fibers. However, inconsistent with our results, the protein expression did not correlate with the mRNA, which was justified as a posttranscriptional modification of the TE. This discrepancy may be because their studies were carried out in cell populations of fibroblasts obtained from pterygium subjected to UV radiation and those of our group were conducted on fresh pathological tissue.

Therefore, in the pathology of pterygium, the protein expression of the mentioned elastic components increases but they do not assemble correctly, thus, producing dysfunctional elastic fibers at the stromal level, which macroscopically and clinically translate into inelastic tissue in its fresh state. This change leads to a loss of functionality that could contribute to the development of other ocular pathologies, such as astigmatism induced by various mechanisms, such as the accumulation of tear film on the leading edge of pterygium or the mechanical traction exerted by it at the level of the cornea [94].

Regarding the expression of FBN1, our results confirmed an increase in mRNA levels in pterygium with respect to the normal conjunctiva at the transcriptional level, although this increase was only discretely significant at the level of protein expression, possibly indicating the existence of messenger degradation or alterations at the translational level.

Other ocular diseases that affect the elastic component, and more specifically the microfibrils of FBN1, include myopia and ectopia lentis; both ophthalmological pathologies are frequently observed in Marfan syndrome, which involves defects in the microfibrils of FBN1. Glaucoma is also associated with this syndrome, although the form of this pathology has not been well characterized [95].

FBLNs are matrix proteins capable of directing the deposition of TE on microfibrils. Different studies have revealed that FBLN4 and FBLN5 were essential for the formation of elastic fibers [67,96], and mutations in both molecules could cause cutis laxa, an inherited disorder associated with degeneration of elastic fibers leading to sagging skin, vascular tortuosity, and pulmonary emphysematous changes [97]. FBLN4 is expressed during early embryogenesis and is necessary for normal vascular, pulmonary, and skin development. Experimental studies on mice lacking FBLN4 have shown that the mice did not form elastic fibers and die perinatally. However, the absence of FBLN5 causes a less severe phenotype, identifying fragmented and irregular elastic fibers in the skin, lungs, and aorta.

Although differences in the distribution of microfibrils have been identified in eye diseases, such as keratoconus [98], limited ophthalmological research has focused on the mechanisms involved in the assembly of elastin, and no studies have directly focused on pterygium. Our group pioneered the analysis of these proteins in pterygium and showed that they have very important roles in the assembly of elastic fibers and participate in various supramolecular structures with binding sites to various proteins, including TE, fibrillin, and proteoglycans.

In our studies, all the FBLNs analyzed, except FBLN4, showed an increase in their protein expression in pterygium as compared with healthy conjunctivae. However, the messenger was only increased in FBLN5, and in the case of FBLN3, the expression even decreased. These results can be explained by the degradation of the mRNA. Thus, protein overexpression may be the result of mechanisms at the posttranscriptional level. However, the results obtained in relation to FBLN4, whose biological role is critical in binding to the LOX enzyme and FBN1 for the development of stable elastin, did not show any type of alteration as compared with the controls. Staying at normal levels could imply the effect of maturation and crosslinking of the rest of the overexpressed components, which could be related to the development of the disease.

Molecular studies have associated fibulin expression with disorders that affect multiple organs, including the eye [99]. These authors demonstrated a significant association between sequence variations in a member of the FBLN gene family and age-related macular degeneration, the most common cause of irreversible vision loss diseases in the developed world [44]. Studies have shown that these proteins were key in stabilizing the structure of the cornea and were synthesized by corneal cells at the epithelium or endothelium level [100].

LOXs are considered to be the main enzymes involved in collagen and elastin crosslinking in the ECM. The most important finding related to LOXs in ophthalmological research is associated with ocular pseudoexfoliation syndrome, which conditions the development and progression of glaucoma [101]. The research results provide evidence of a primary alteration in the LOXL1 gene (polymorphisms rs1048661 and rs3825942) that constitutes a risk factor involved in the alteration of elastic fiber homeostasis. Among the genetic factors, LOXL1 polymorphism constitutes the main genetic risk identified for the development of the disease [102]. In fact, it has been reported that LOXL1 knockout mice developed ocular pseudoexfoliation syndrome traits, which related decreased enzyme activity to a predisposition to the disease [103]. In addition, exposing cultures obtained from Tenon’s capsule fibroblasts with high- and low-risk haplotypes of LOXL1 to factors such as ultraviolet radiation, hypoxia, oxidative stress states, or TGF-β has been shown to produce a significant increase in the expression levels of LOXL1 proteins and other elastin constituents, such as FBN1 and FBLN4. Therefore, it has been postulated that genetic factors in combination with other factors, particularly TGF-β activity and oxidative stress, could cooperate in the development of pseudoexfoliation syndrome [104,105,106].

According to studies of LOX and LOXL1, other related components in elastogenesis and ocular exfoliation syndrome have recently been reported. In fact, the presence of FBLN5 polymorphisms rs7149187: G > A and rs929608: T > C has been associated with high-risk variants in the development of the disease [107]. Therefore, the study of the components involved in the synthesis of elastin is an area of great interest in ophthalmological research.

Gene expression analyses have shown clear differences between primary pterygium and healthy conjunctiva [108]. Among the positively regulated genes, some encoded proteins involved in wound healing and components of the ECM, including different types of collagens, LOXL1, and various structural proteins. This was consistent with our RT-PCR results that showed a significant increase in LOXL1 mRNA in disease that was associated with a corresponding level of protein overexpression. 

In our case, overexpressed LOXL1 mRNA and protein levels were identified in pterygium, but, in the case of LOX, the messenger remained stable and only the protein levels showed a significant increase in pterygium pathology. Related to this last result, we must remember that a selective role for LOXL1 has been proposed in elastin but not in collagen metabolism based on desmosine and hydroxyproline levels, which represent elastin and collagen crosslinks, respectively. The authors of one study reported significantly lower desmosine levels in various tissues with mutated LOXL1, while hydroxyproline levels remained unchanged. This apparently showed that one of the main substrates of LOX was collagen I. However, LOXL1, but not LOX, was specifically targeted to elastogenesis sites [72], showing that LOXL1 was closely related to elastic fibers, while LOX is more widely distributed.

Recently, transcriptional profiling to identify the key genes and pathways of pterygium and transcriptome analysis of mRNAs have been performed, indicating that differentially expressed RNAs were associated with ECM organization, blood vessel morphogenesis, and focal adhesion and that the upregulated genes were mainly associated with the ECM, cell adhesion, or migration [109,110].

In summary, taking into consideration all the studies carried out by our research group on the pathogenesis of pterygium throughout our scientific career, we can establish that the changes in the fibroelastic component of the ECM that occur in pterygium are based on the following:

- Increased synthesis and deposition of collagen fibers favor the immature form of collagen type III, and thus show a process of tissue remodeling;
- Increased protein levels in most of the constituents necessary for the development of elastic fibers, except FBLN4, whose biological roles are critical in the binding of the enzyme LOX and FBN1 for the development of stable elastin;
- Gene overexpression of TE, FBN1, FBLN5, and LOXL1, while the expression levels of LOX, as well as FBLN2 and -4, are comparable to those of controls.

Future research in this regard is strongly recommended, since, in our opinion, the FBLN4 and the LOX protein family should be considered to be important targets for the development of future therapies for treating diseases involving remodeling of extracellular matrix.

## 8. Conclusions

In conclusion, we can affirm that the two most important fibrillar proteins of the ECM of the conjunctival stroma, collagen, and elastin, as well as several constituents involved in elastic fiber assembly are overexpressed in human pterygium; thus, supporting the hypothesis that there is dysregulation in the synthesis and crosslinking of the fibroelastic component, constituting an important pathogenetic mechanism for the development of the disease.

## Data Availability

Not applicable.
